# Wine Allergy in a Wine-Growing District: *Tolerance Induction in a Patient With Allergy to Grape Lipid-Transfer Protein*

**DOI:** 10.1097/WOX.0b013e3181c82113

**Published:** 2010-01-15

**Authors:** Susanne G Schäd, Jiri Trcka, Iris Lauer, Stephan Scheurer, Axel Trautmann

**Affiliations:** 1 The Department of Dermatology and Venereology, University of Rostock, Rostock, Germany; Division of Allergology, Paul-Ehrlich-Institut, Langen, Germany; Department of Dermatology, Venereology, and Allergology, University of Würzburg, Würzburg, Germany

**Keywords:** specific oral tolerance induction, grape allergy, wine allergy, food allergy, anaphylaxis, lipid-transfer protein

## Abstract

**Background**

An IgE-mediated allergy against a lipid-transfer protein of grapes was the cause of repeated severe anaphylaxis in a patient after consumption of grapes, wine, and raisins.

**Objective**

Although the patient was aware of her grape allergy, avoidance proved difficult and accidental anaphylaxis occurred. Furthermore, wine allergy in a wine-growing district means a non-negligible restriction of quality of life.

**Methods**

Although there is little data on specific oral tolerance induction (SOTI) in lipid-transfer protein (LTP) allergy, SOTI with increasing doses starting from approximately 20 mg of grapes was done. For follow-up, skin tests, grape-specific IgE and IgG4, basophil activation tests, and immunoblotting were performed.

**Results**

Within 3 days the patient reached tolerance to the daily maintenance dose of 20 g of grapes (about 3 grape pieces) without anaphylaxis symptoms. Two months later, a controlled challenge with a total of 66.5 mL of white wine was tolerated. Grape-specific IgE stayed stable at 2.37 kU/L (class 2) and grape-specific IgG4 was first detectable 21 months after SOTI. Prick-to-prick skin tests continued to be positive to grapes, to raisins, and to white and red wine. The basophil activation test still showed strong IgE-mediated activation of basophils after stimulation with grape extract. Immunoblotting still detected IgE binding to a 8-kDa protein.

**Conclusions**

We performed SOTI in a patient with severe IgE-mediated allergy against the LTP Vit v 1 of grapes and reduced the risk of anaphylaxis because of accidental intake of any kind of grapes. However, underlying mechanisms of SOTI and maintenance of the established tolerance are still not known.

## Introduction

Allergy to grapes has been rarely reported despite grapes being widely consumed as fresh fruit, juice, and wine[1-3]. The major allergens of grape and wine have been identified as endochitinase 4A, a lipid-transfer protein (LTP), and a thaumatin-like protein[4]. Although the majority of infants and young children outgrow their allergy to, for example, cow's milk and hen's egg, food allergy in adults to, for example, fruits such as grapes typically persists for a lifetime[5]. In routine clinical settings treatment for IgE-mediated grape allergy is avoidance of grapes with adequate pharmacotherapy in the event of accidental ingestion[6]. Strict avoidance of any kind of grapes is rather difficult and may lead to severe anaphylaxis in the case of dietary failure. Interestingly, many of the patients who experience life-threatening food-induced anaphylaxis are aware of their food allergies. In a study conducted in the United States, allergy to the triggering food was known in 41% of emergency room visits for allergic reactions to foods[7]. This highlights the search for additional therapeutic strategies beneath the importance of allergen avoidance.

Subcutaneous injection immunotherapy is not recommended for food allergy because of the high incidence of systemic adverse reactions[8-10]. Achieving tolerance by specific oral tolerance induction (SOTI) is a promising treatment option in patients with food allergy, but is still controversial and no standardized protocols are yet available. During SOTI, the offending food is administered orally, starting with very low doses, followed by a steady increase up to an amount equivalent to a usual daily oral intake. Successful SOTI was repeatedly reported with cow's milk and hen's egg, which are not LTP allergens. Thus far, Enrique et al [11] published a randomized, double-blind, placebo-controlled study of patients allergic to Spanish hazelnut who were treated successfully by an oral/sublingual immunotherapy with standardized hazelnut extract. Major allergens in these patients were identified as Cor a 1, a protein homologous to the Bet v 1 allergen, and Cor a 8, a hazelnut lipid-transfer protein, not associated with birch pollen allergy[12].

Here, we report successful SOTI in a patient with IgE-mediated allergy against a LTP of grapes, identified as Vit v 1 (*Vitis vinifera *= grape wine, botanical family Vitaceae)[13]. The results of in vivo and in vitro diagnostic procedures before and after SOTI demonstrated, on the one hand, unchanged IgE-mediated sensitization, and on the other hand, SOTI-induced grape-specific IgG_4 _antibodies.

## Methods

### Patient

We recently reported on a now 34-year-old German woman who had severe anaphylactic reactions after consumption of about 100 mL of wine, 5 pieces of fresh white or blue grapes, and 3 pieces of raisins. Her last episode with palmoplantar pruritus, angioedema of the lips and tongue, dyspnea, dysphagia, and tachycardia was 7 years ago, 60 minutes after eating about 3 pieces of raisins. Shortly after this last episode, our study could demonstrate that her anaphylactic symptoms were due to an IgE-mediated allergy against the LTP Vit v 1 of grapes without associated pollinosis[13]. She had to avoid all kinds of grapes, raisins, grape juice, wine, and champagne for 3 years before specific oral tolerance induction was performed.

### SOTI

General principles of our SOTI protocol are as follows: (a) the time interval between SOTI and anaphylaxis symptoms has to be at least 4 weeks; (b) during the entire SOTI the patient is monitored and equipment for emergency treatment is available; (c) the dose increases stepwise in 3 consecutive days to the maintenance dose; (d) absolute and relative contraindications for SOTI are strictly adhered to; (e) before SOTI written informed consent is obtained. The schedule consisted of administering increasing amounts of white grapes starting from 19.5 mg of grapes ground in a mortar and diluted with 10 mL of 0.9% sodium chloride (Table 1). The grape amount was doubled every 30 minutes to 625 mg of grapes on the first day. On the second day, the dose was continuously doubled every 30 minutes starting from the last dilution on the day before up to 20 g of grapes diluted with 10 mL of 0.9% sodium chloride. On day 3 the maximum dose of 3 pieces of white grapes (20 g) was given.

**Table 1:** SOTI Protocol for Grape Allergy

Parameter	SOTI procedure
Starting dose of grape	10 mL of 20 g of grapes diluted 1:1024 with 0.9% sodium chloride, corresponding to 19.5 mg of grapes
Time between steps	30 minutes
Increment of steps	Doubling doses
Number of steps	14
Time for whole procedure	3 days
Maximal dose	20 g of grapes

### Challenge test

Two months later, a blinded, not placebo-controlled oral challenge test was performed starting from 0.5 mL of undiluted white wine. The dose was then increased every 30 minutes to 1.0, 5.0, 10, and 50 mL.

### Skin tests

Prick-to-prick tests with white and blue grapes, raisins, and white and red wine were performed before and 3 months after successful SOTI. The prick-to-prick tests were done as previously described[13]. The test was defined as "1+" positive reaction with a wheal diameter of 2-4 mm, "2+" positive reaction with a wheal diameter of 4-6 mm, and "3+" positive reaction with a wheal diameter more than 6 mm. All tests were performed according to the EAACI recommendations [14].

### Grape-Specific IgE/IgG_4_

Serum was analyzed for grape-specific IgE and specific IgG_4 _antibodies (f259) by the Phadia CAP System (Phadia AB, Uppsala, Sweden) according to the manufacturer's instructions.

### Grape extract

Protein extract was prepared from fresh white grapes by a low-temperature acetone powder method as previously described[13].

### Basophil activation test

The basophil activation test (BAT) was performed as previously described[13]. Blood (5 mL) from the patient and a control (nonallergic) person was used within 6 hours of blood sampling for the BAT, which is based on the in vitro allergen-induced activation of basophils. The assay was performed using a kit according to the manufacturer's instructions (Bühlmann Laboratories, Basel, Switzerland). Briefly, leukocytes were stimulated in vitro with grape extract and commercial grape antigen (555; Allergopharma, Freiburg, Germany) at concentrations ranging from 10^-8 ^to 1 *μ*g/mL and from 0.5 × 10^-9 ^to 0.5 *μ*g/ml, respectively, control antigen (yellow jacket), and positive control (activating anti-Fc*ε*RI antibody). The cells were double-stained with anti-CD63-Phycoerythrin and anti-IgE-fluorescein isothiocyanate-labeled antibodies. Activated basophils (CD63^+ ^and IgE^+ ^double-positive cells) were counted by flow cytometry at 488 nm on a FACSCalibur (Becton Dickinson Immunocytometry Systems, Mansfield, Mass., USA) using Cell Quest Software.

### Immunoblotting

Briefly, grape extract (70 *μ*g of protein per cm) was separated by SDS-PAGE (17.5%) according to Lämmli under nonreducing conditions and electroblotted onto nitrocellulose membranes (0.2 mm; Schleicher und Schüll, Dassel, Germany) (400 mA, 50 minutes) blocked in Tris-buffered saline/0.3% Tween 20 (Sigma-Aldrich, Steinheim, Germany). Transfer was controlled by reversible staining of membranes with Ponceau S (Sigma Diagnostics, St. Louis, USA). Membranes were incubated with the patient's 1:7 diluted sera and bound IgE antibodies were detected with mouse-antihuman IgE-biotin (1:1.500; KPL, Gaithersburg, Md, USA) followed by streptavidin-AP (1:3.000; Caltag, Burlingame, Calif., USA). Bound antibodies were visualized with nitroblue tetrazolium/5-bromo-4-chloro-3-indolyl phosphate as substrate in 0.1 M Tris-buffered saline, pH 9.5, according to the manufacturer's instructions (Biorad, Munich, Germany).

## Results

### SOTI and challenge test

After successful completion of SOTI without any symptoms, the patient maintained the achieved tolerance by a daily maintenance dose of 20 g of white or blue grapes (about 3 pieces of grape). Two months after SOTI, she tolerated a challenge test with a total dose of 66.5 mL of white wine. So far, 4 years after SOTI, no anaphylaxis symptoms occurred through accidental intake of any kind of grapes. A double-blind, placebo-controlled oral grape challenge further testing tolerance was refused by the patient because of considerable fear of anaphylaxis symptoms.

### Skin test and grape-specific IgE/IgG_4_

Prick-to-prick tests revealed positive reactions to fresh white (1+) [before SOTI 2+] and blue grapes (1+) [2+], to raisins (2+) [3+], and to white (1+) [1+] and red wine (1+) [2+]. Specific IgE to grape was 2.37 kU/L (class 2) 2.43 kU/L, class 2] and specific IgG_4 _to grape was 160 *μ*gA (assay-specific)/L 21 months after SOTI [not detectable before SOTI].

### BAT

Activation of the patient's basophils after stimulation with self-prepared grape extract at different concentrations yielded 93.0% and 89.7% activated basophils at 0.1 *μ*g/mL and 10^-5 ^*μ*g/ml, respectively, and with commercial grape antigen 89.1% and 17.4% activated basophils at 0.5 × 10^-1 ^and 0.5 × 10^-2 ^*μ*g/mL, respectively. Background of the negative control was measured as 9.6%. In grape extract- and commercial grape antigen-stimulated samples of the control person, no basophil activation was observed, that is, 5.7% activated basophils at 0.1 *μ*g/mL of grape extract and 5.1% activated basophils at 0.5 × 10^-1 ^*μ*g/mL of commercial grape antigen. In both the patient and control person, stimulation with control antigen (yellow jacket) was negative (2.9% and 8.0%), whereas positive controls using activating anti-Fc*ε*RI antibody confirmed basophil activation (93.0% and 92.1%).

### Immunoblotting

**Figure 1:**
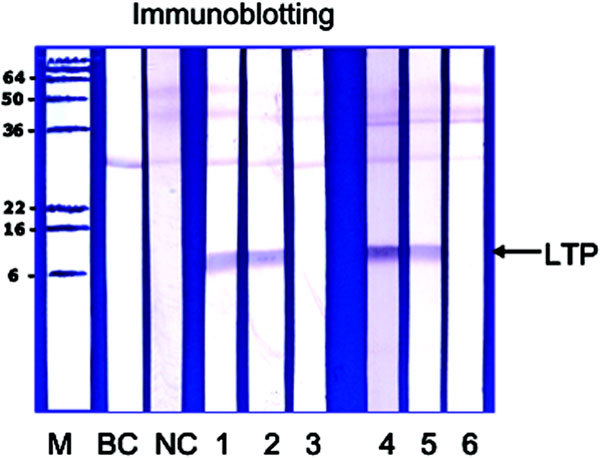
**Immunoblotting**. Lane M, molecular-weight marker proteins; lane BC, buffer control; lane NC, nonallergic control; lane 1, IgE binding of patient allergic to wine; lane 2, preincubation of patient's serum with 15 *μ*g of BSA; lane 3, preincubation of patient's serum with 15 *μ*g of purified recombinant cherry LTP Pru av 3; lanes 4-6, positive control serum (patient from Spain with known IgE antibody reactivity to different LTPs: lane 4, without inhibitor; lane 5, preincubation with 15 *μ*g of BSA; lane 6, preincubation with 15 *μ*g of rPru av 3).

Before and after SOTI grape-specific IgE was further analyzed by immunoblotting using the self-prepared grape extract. Figure 1 showed only one single IgE-reactive band with an apparent molecular weight of 8 kDa (lanes 1 and 2), whereas for the nonallergic control no IgE antibody reactivity was detectable (lane NC). The IgE reactive band was completely inhibited by preincubation with 15 *μ*g of cherry LTP (lane 3)--no inhibition with bovine serum albumin as control (lane 2)--suggesting that this band represents the LTP Vit v 1. In a comparison of immunoblotting experiments before and after SOTI, no significant reduction of IgE-antibody reactivity to the grape LTP could be observed [13].

## Discussion

The mainstay in the management of IgE-mediated food allergy is strict avoidance of exposure to the offending allergen[15]. However, this approach can be difficult in the case of common food or when the allergen is hidden and not necessarily labeled in commercial food. It should always be remembered that many of the most serious allergic reactions occur in restaurants and other food-service establishments where full-label disclosure of ingredients is typically not practiced[10,16]. Furthermore, the elimination diet may reduce quality of life and can induce eating disorders with psychological problems.

Given the high incidence of systemic reactions using subcutaneous immunotherapy for IgE-mediated peanut allergy,[17] oral immunotherapy has been investigated as an alternative during the past few years. Our patient suffered from repeated IgE-mediated anaphylaxis after ingestion of very small amounts of any kind of grapes. She lives in a wine-growing district where numerous festivities take place in a given year concerning the wine cultivated there. Furthermore, she takes vacations regularly in Italy and enjoys Mediterranean kitchen. Therefore, the strict avoidance of wine was in her case accompanied by a substantial restriction of her quality of life and we decided to perform SOTI. Little is known about SOTI in patients with IgE-mediated LTP allergy. Twenty-three patients from Spain with an IgE-mediated allergy to a Bet v 1-homologous hazelnut protein or to a hazelnut LTP were treated with oral/sublingual immunotherapy of a standardized hazelnut extract or with a saline solution as placebo in a randomized, double-blind, placebo-controlled study[11]. For the active specific immunotherapy group of 12 patients, the starting dose concentration was 2 × 10^-11 ^mg and the final dose was 119.51 mg of hazelnut protein. With a rushed schedule, the build-up phase was completed in 4 days. Thereafter, mean hazelnut quantity provoking objective symptoms increased from 2.29 to 11.56 g in the active group versus 3.49 to 4.14 g in the placebo group.

More experiences exist for SOTI with cow's milk, mostly in children[18,20]. Meglio et al [20] reported a completely successful SOTI with cow's milk in 15 of 21 children (71.4%). Although 8 of these children showed no allergic reactions reaching the full cow's milk intake, 7 children presented some, mostly temporary symptoms such as moderate asthma, throat pruritus, urticaria, rhinitis, abdominal and epigastric pain, and vomiting. In the study by Patriarca et al, [18] cow's milk SOTI was successful in 4 of 6 children. In a preliminary report on 3 patients with allergy to cow's milk or hen's egg, all 3 patients reached tolerance to the maximum dose after 37, 41, and 52 weeks[21]. Recently, a 24-month egg SOTI for children with nonanaphylactic egg allergy resulted in increased tolerance to egg upon placebo-controlled challenges to levels higher than those found in accidental ingestions[22]. During SOTI few patients cannot achieve the full maintenance dose because of anaphylaxis symptoms such as urticaria, angioedema, abdominal pain, and hypotension [20,23].

However, in the majority of cases these adverse reactions can be controlled by oral antihistamines [20].

So far, no standardized protocols for SOTI are available. There are different regimens for SOTI with cow's milk reported: An ultrarush regimen starts with a low dose followed by several steps usually every 30 minutes with a half-logarithmic increasing dose up to 3 to 5 g of protein, resulting in an overall time of 4 to 5 hours[24]. A conventional SOTI procedure foresees the cow's milk administration at home with the exception of the first doses. It starts with a very low dose followed by less than doubling doses every 24 hours. In 2 to 3 months the maximum dose of 3 to 5 g of protein can be achieved[21]. A rush protocol of SOTI starts with a low dose followed by doubling doses every 2 hours. This rush-SOTI with a maximum dose of 3 to 5 g of cow's milk protein takes approximately 1 week [19].

Regarding total time required to reach the maintenance dose, we used a SOTI regimen modified between ultrarush and rush. The dose of 3 pieces of whole grapes (~20 g) was achieved within 3 days and is now continued as the daily maintenance dose. No clinical symptoms occurred during SOTI. After 2 months of the maintenance phase, an oral wine challenge test was also tolerated. Until now, 4 years after SOTI and continous daily grape intake of the maintenance dose, the patient seems to be also protected against reactions after accidental ingestion of any kind of grapes. Her quality of life clearly improved as she can now drink small amounts of wine (up to 66.5 mL) and safely eats, including processed foods and those eaten outside the home.

SOTI may induce tolerance which persists a lifetime, but it is not clear whether the maintenance of the established tolerance is dependent on continuous allergen intake[20,23]. Rolinck-Werninghaus et al [21] reported moderate systemic allergic reactions in all 3 patients after re-exposure to the allergen when maintenance treatment was stopped. The acquired tolerance during/after SOTI may reflect the natural course of the allergic disease over time or may be due to a specific immune modulation by the SOTI procedure. Animal studies demonstrated that the induction of anergy or deletion of allergen-specific T cells and the activation of regulatory T cells may be 2 possible mechanisms for achieving oral tolerance[25]. Therefore, Niggemann et al [26] proposed the term "specific oral tolerance induction" for this treatment instead of oral immunotherapy, oral desensitization, or oral hyposensitization. Few studies in humans showed significant decrease of allergen-specific IgE after 6 months and significant increase of allergen-specific IgG_4 _18 months later[23]. In the placebo-controlled study of Enrique et al [11] laboratory data demonstrated an increase in IgG_4 _and IL-10 levels after immunotherapy in the actively treated group. Our patient demonstrated grape-specific IgG_4 _for the first time 21 months after SOTI while her specific IgE remained stable. However, the precise mechanisms for oral tolerance induction in humans still have to be clarified[27]. In our investigation, prick-to-prick skin tests, the in vitro BAT, and the immunoblotting confirmed despite clinical tolerance the presence of IgE against the LTP Vit v 1 of grapes, reflecting at least persisting sensitization.

In conclusion, our study demonstrates that SOTI can be a successful treatment option in patients with IgE-mediated LTP allergy by reducing the risk of anaphylaxis and by increasing the patient's quality of life. Further studies are necessary to determine the indications, protocol regimen, time frames, immunologic changes, and the transiency of the effect of SOTI in LTP allergy.
